# Long-Term Socioeconomic and Mental Health Changes After Out-of-Hospital Cardiac Arrest in Women and Men

**DOI:** 10.1161/CIRCOUTCOMES.124.011072

**Published:** 2024-07-08

**Authors:** Robin L.A. Smits, Fleur Heuvelman, Karen Nieuwenhuijsen, Patrick Schober, Hanno L. Tan, Irene G.M. van Valkengoed

**Affiliations:** Department of Public and Occupational Health (R.L.A.S., I.G.M.V.), Amsterdam UMC location University of Amsterdam, Amsterdam Public Health Research Institute, The Netherlands.; Department of Epidemiology and Data Science (F.H.), Amsterdam UMC location University of Amsterdam, Amsterdam Public Health Research Institute, The Netherlands.; Department of Public and Occupational Health, Amsterdam UMC location University of Amsterdam, Coronel Institute of Occupational Health, Amsterdam Public Health Research Institute, The Netherlands (K.N.).; Department of Anesthesiology, Amsterdam UMC location Vrije Universiteit Amsterdam, The Netherlands (P.S.).; Department of Clinical and Experimental Cardiology, Amsterdam UMC location University of Amsterdam, Heart Centre, Amsterdam Cardiovascular Sciences, The Netherlands (H.L.T.).; Netherlands Heart Institute, Utrecht (H.L.T.).

**Keywords:** men, mental health, socioeconomic factors, survivors ◼ women

## Abstract

**BACKGROUND:**

Long-term effects of out-of-hospital cardiac arrest (OHCA) may affect the ability to work and mental health. Our aim was to analyze 5-year changes in socioeconomic and mental health outcomes after OHCA in women and men.

**METHODS:**

We included 259 women and 996 men from North Holland, the Netherlands, who survived 30 days after OHCA occurred between 2009 and 2015. We assessed changes in employment, income, primary earner status, and anxiety/depression (using medication proxies) from the year before the OHCA to 5 years after with generalized linear mixed models, stratified by sex. We tested differences in changes by sex with interaction terms. Additionally, we explored yearly changes. The 5-year changes after OHCA were compared with changes in a sex- and age-matched sample of people without OHCA. Differences were tested using an interaction term of time and OHCA status.

**RESULTS:**

In both women and men (median age [Q1, Q3]: 51 [45, 55] and 54 [48, 57] years, respectively), decreases from before OHCA to 5 years thereafter were observed in the proportion employed (from 72.8% to 53.4% [women] and 80.9% to 63.7% [men]) and the median income. No change in primary earner status was observed in either sex. Dispensing of anxiety/depression medication increased only in women, especially after 1 year (odds ratio, 5.68 [95% CI, 2.05–15.74]) and 5 years (odds ratio, 5.73 [95% CI, 1.88–17.53]). Notable differences between women and men were observed for changes in primary earner status and anxiety/depression medication (eg, at year 1, odds ratio for women, 6.71 [95% CI, 1.96–23.01]; and for men, 0.69 [95% CI, 0.33–1.45]). However, except for anxiety/depression medication in women, similar changes were also observed in the general population.

**CONCLUSIONS:**

OHCA survivors experience changes in employment, income, and primary earner status similar to the general population. However, women who survived OHCA more often received anxiety/depression medication in the years following OHCA.

**WHAT IS KNOWN**

- Research on long-term (≥1 year) changes in socioeconomic factors and mental health following out-of-hospital cardiac arrest (OHCA) is limited.
- Most studies do not address potential disparities in long-term consequences between women and men.
- Evidence on long-term changes as compared with the general population is limited.

**WHAT THE STUDY ADDS**

- Employment, income, and primary earner rates change over 5 years after OHCA in women and men who survived OHCA, but changes are similar to those observed in the general population.
- Women who survived OHCA experienced an increase in anxiety/depression medication in the 5 years following OHCA, which was not observed in the general population.

While mortality rates of out-of-hospital cardiac arrest (OHCA) remain high,^1^ survival rates have increased globally over the past decades.^2^ The rising number of survivors necessitates an understanding of the long-term consequences they face.^3^ Survivors have to accept potential changes in their new life and adapt mentally, physically, and socially.^4^ High rates of anxiety, depression, and posttraumatic stress disorder are reported.^5^ Moreover, many struggle to return to work, potentially affecting their social position and income.^6^ These factors can impact overall quality of life^7,8^ and physical health.^9–11^

Although short-term (<1 year) outcomes have been extensively reported and qualitative studies indicate substantial challenges for OHCA survivors,^12^ quantitative research on long-term (≥1 year) changes in socioeconomic factors and mental health following OHCA is limited.^6,13,14^ Studies suggest that, although return to work increased from the first year post-OHCA onward, up to 25% of individuals did not return even after several years.^6,13^ While this may be by choice, it is likely to affect income and relate to mental health. Substantial rates of anxiety and depression that persist over time were reported.^14^ However, these studies lack comparisons with the general population and do not address potential disparities in long-term consequences between women and men.

This is relevant, as the long-term effects of an OHCA may vary between women and men. Not only do sex differences exist in short-term survival, but women also have a poorer neurological outcome after OHCA.^15^ Additionally, women suffering from OHCA are generally older and fulfill different societal roles in life than men (eg, more caregiving activities). Indeed, the sparse literature suggests that being a woman seems to be an unfavorable predictor of return to work,^6,16^ and women tend to report a higher prevalence of anxiety after 3 months than men.^17^ Thus, long-term changes in socioeconomic and mental health outcomes following OHCA may differ by sex.

It is crucial to identify changes in the lives of women and men who survived OHCA to be able to adopt a patient-oriented approach that goes beyond a focus on mere survival. Gaining insights into long-term changes in both socioeconomic and mental health factors can assist caregivers, patients, and their families in comprehending the lasting impact of OHCA on daily life, enabling optimization or the development of tailored treatment strategies. The aim of this study was to examine changes over 5 years in measures reflecting socioeconomic position and mental health, specifically employment status, primary income, primary earner status, and anxiety/depression (assessed by dispensing records of anxiety/depression medication) after OHCA in men and women. We compared changes by sex and determined how changes differed from those in the general population.

## METHODS

### Data Availability

The results are based on calculations by the Amsterdam Medical Centre using nonpublic microdata from Statistics Netherlands. Under certain conditions, these microdata are accessible for statistical and scientific research. For further information, email microdata@cbs.nl.

### Data Sources

Data on emergency medical services-attended OHCAs between 2009 and 2015 were included from the Amsterdam Resuscitation Studies registry, a prospective registry of consecutive all-cause OHCA cases in 1 contiguous region of the Netherlands (North Holland province), including urban and rural areas and containing 2.4 million inhabitants.^18^ Extensive collaboration with all emergency medical services in this study region resulted in capturing over 95% of all emergency medical services-attended OHCA attempts, minimizing inclusion bias. Written, informed consent was obtained from all surviving participants. The medical ethics review board of the Academic Medical Centre of the University of Amsterdam approved the study, including the use of data from patients who did not survive the OHCA. To augment these data with socioeconomic (employment status, income, and primary earner status) and medication data (Anatomical Therapeutic Chemical codes), the data from the Amsterdam Resuscitation Studies were linked to individual-level data from Statistics Netherlands (a Dutch government agency collecting national statistical data) with algorithmic deterministic linkage performed by Statistics Netherlands. The linkage was based on (1) date of birth and sex, and (2) postal code, house number, and date at which the individual lived at the address. Few cases could not be linked due to incorrect address information; ≈96% of OHCA cases were successfully matched. Unmatched cases could not be included in our study. After linkage, the cases were anonymized by Statistics Netherlands.

### Study Population

Patients who survived for at least 30 days and were aged ≥25 years (assuming financial independence from this age onward)^19^ were included in the study. We excluded OHCAs from clear noncardiac causes (eg, drug overdose, trauma, and asphyxiation). If a patient experienced multiple OHCAs, only the first event was considered. Of the 5988 people who suffered an OHCA, 1255 survived until hospital discharge (Figure S1). Of all these individuals, data were available for analyses of anxiety/depression medication. For socioeconomic variables (ie, employment status, income, and primary earner status), the analyses were restricted to individuals aged ≤60 years, as the legal retirement age was ≈65 years in the Netherlands during the study period. This reduced the influence of retirement on employment status. Additionally, only those cohabiting at baseline were analyzed for primary earner status, as individuals in single-person households were always categorized as the primary earner. Thus, data from 509 individuals were used for analyses of employment and income, and 409 individuals for primary earner status analysis. In addition, a comparison population (henceforth: general population) was selected within Statistics Netherlands using 2:1 sex- and age-matching from residents without OHCA in the Amsterdam Resuscitation Studies study region (Figure S1).

### Outcomes

The baseline for all primary outcome measures was the year preceding OHCA, as data were reported annually. A sensitivity analysis defined baseline as 2 years before OHCA. Outcomes were determined 1 to 5 years after the year of OHCA, with change over 5 years as the main outcome.

Employment status, income, and primary earner status were classified based on primary income, reflecting gross income from labor and self-employment, obtained from tax authorities. Individuals with zero primary income were classified as unemployed, and individuals with an income above zero or below zero (in the case of self-employment where the company reported a loss) were classified as employed. Income changes were assessed using the primary income as a continuous variable. Primary earner status reflects economic responsibility and was based on the individual’s contribution to the total household income, where the primary earner had the largest contribution to the total household income. The presence of anxiety and/or depression (henceforth: anxiety/depression medication) was classified from the presence/absence of drug dispensing records at any public pharmacy in the Netherlands with Anatomical Therapeutic Chemical codes N06A (anti-depressants) or N05B (anxiolytics).

### Other Variables

Sex was based on the assigned sex at birth from the population register. Since we are aware of the fact that differences observed in our study may not only reflect biological factors (sex) as suggested by our choice of the term sex but also sociocultural factors (gender), we chose to use the terms “women” and “men” over the use of the terms male and female. The age at the time of OHCA was determined from the ambulance dispatch form. We included standardized disposable household income (henceforth: household income) for the year before the OHCA, divided into tertiles, as a measure of an individual’s overall financial situation. Household income is a potential explanatory factor for sex differences, as it may differ between women and men and is associated with health.^20–23^ To describe our study population, we also reported whether people received disability insurance, unemployment, or retirement benefits as determined by Statistics Netherlands based on income sources for that year. Cohabiting status was based on the number of adults in the household, regardless of relationship status, and classified as no (living alone) or yes (living together with at least one other adult). In addition, based on whether people were living at home or in an institution (eg, a nursing home), they were classified as living at home or not living at home. Lastly, we reported marital status classified as married or not married (including widowed and divorced).

### Statistical Analysis

Baseline characteristics were described for women and men from the overall study population and those aged <60 years. We determined proportions of employment, primary earner status (only in those cohabiting), and anxiety/depression medication, as well as the median income at baseline and annually for the 5 follow-up years, stratified by sex. To examine changes over time in the outcomes, we performed sex stratified age-adjusted analyses with generalized linear mixed models with a logit link function for dichotomous outcomes and an identity link function for continuous outcomes.^24^ In these analyses, we included the ID of the OHCA survivor as a random effect and year and age as fixed effects. Year was included as a categorical variable to indicate the time point of the outcome measure, with baseline as the reference category. The 5-year change in the OHCA population was considered our primary outcome. The other analyses were considered additional analyses as they were included to facilitate the interpretation of our findings.^25^ For each of the 4 outcomes, we assessed whether the changes in outcomes differed over time between women and men by adding sex and an interaction term for sex and year as fixed effects to the model, applied to the population including both women and men. In the case of a statistically significant interaction, we explored whether differences between women and men over time were explained by sex differences in household income at baseline by adding household income to the interaction model (change in beta estimate of the interaction by >10%).^26^

To determine whether the observed changes over time were likely related to OHCA or a reflection of trends in the general population, analyses were repeated within a general population sample (see the Study Population section). Differences in outcomes over time between the OHCA population and the general population were formally tested by adding an interaction term of OHCA status and time.

We performed several sensitivity analyses to assess the robustness of our findings. First, generalized estimating equations analyses with robust standard errors using the exchangeable correlation structure for all outcome variables are an alternative to generalized linear mixed models for dealing with nonindependent data.^27^ Therefore, we opted for this analysis, with an adjustment for age, as a sensitivity analysis. Additionally, baseline values were determined 2 years before OHCA instead of 1 year before to assess whether our baseline estimate had potentially been affected by changes in health status just before OHCA.

Statistical analyses were performed in RStudio (The R foundation), version 4.0.3. All tests were 2-sided, and statistical significance was defined as *P*<0.05.

## RESULTS

The median age of women and men in the total OHCA study population was 63 years (interquartile range, 52, 73) and 63 years (interquartile range, 55, 71), respectively. In the population under 60 years, this was 51 (interquartile range, 45, 55) and 53.5 (interquartile range, 48, 57; Table 1). Compared with men, women <60 years were less often employed, the primary earner of the household, or cohabiting or married in the year before OHCA. Women also had a lower median income and a higher prevalence of anxiety/depression medication at baseline.

**Table 1.**
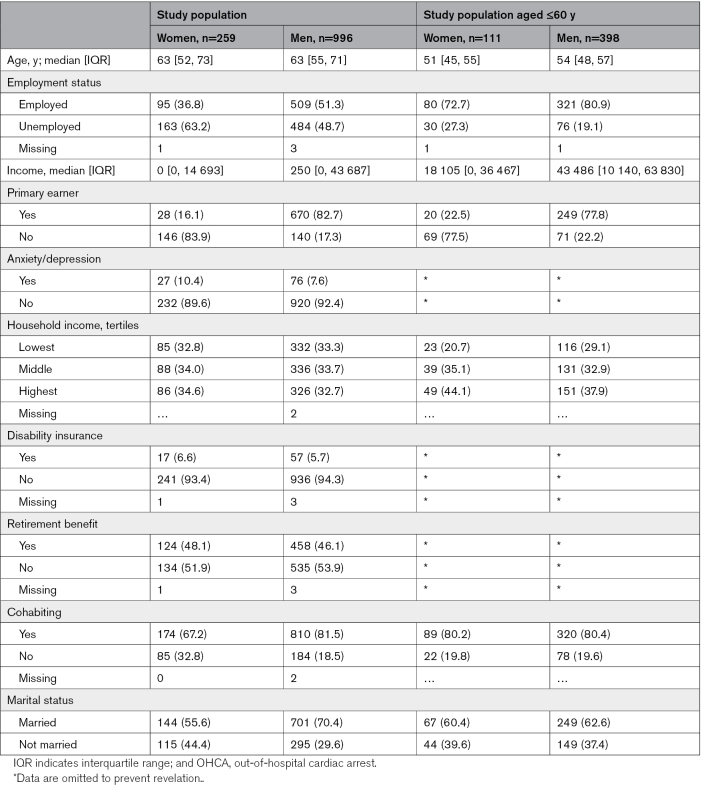
Baseline Characteristics in the Year Before OHCA Overall, and Stratified by Sex

The Figure presents crude changes in outcome variables over time for both the OHCA population and the matched general population. From baseline to 5 years after OHCA, employment rates declined from 72.8% to 53.4% in women and from 80.9% to 53.7% in men (Table S1). Accordingly, median income declined steadily from 1 to 5 years after OHCA for both sexes. These trends remained after adjustment for age (Table 2). Formal interaction testing did not indicate significant differences in the changes over time between women and men. Yet, for primary earner status, we observed differing patterns between women and men, with a small but not statistically significant increase in the proportion of primary earners in women (22.5%–27.4%) and a decrease in men (77.8%–76.7%; *P* value for interaction=0.01). Use of anxiety/depression medication increased significantly in women, particularly in the first year after OHCA, but this trend was not observed in men (*P* value for interaction=0.04). The difference in changes in primary earner status was not explained by sex differences in household income. For anxiety/depression medication, the difference was partly explained by household income, as reflected by a change in beta estimate >10% (Table S2).

**Table 2.**
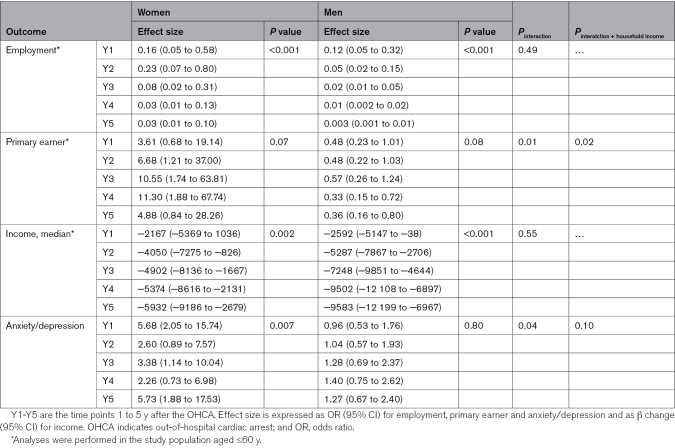
Changes in Outcomes Over Time in the OHCA Population Compared With Baseline by the Generalized Linear Mixed Model, Stratified by Sex, and Interaction Between Sex and Time

**Figure.**
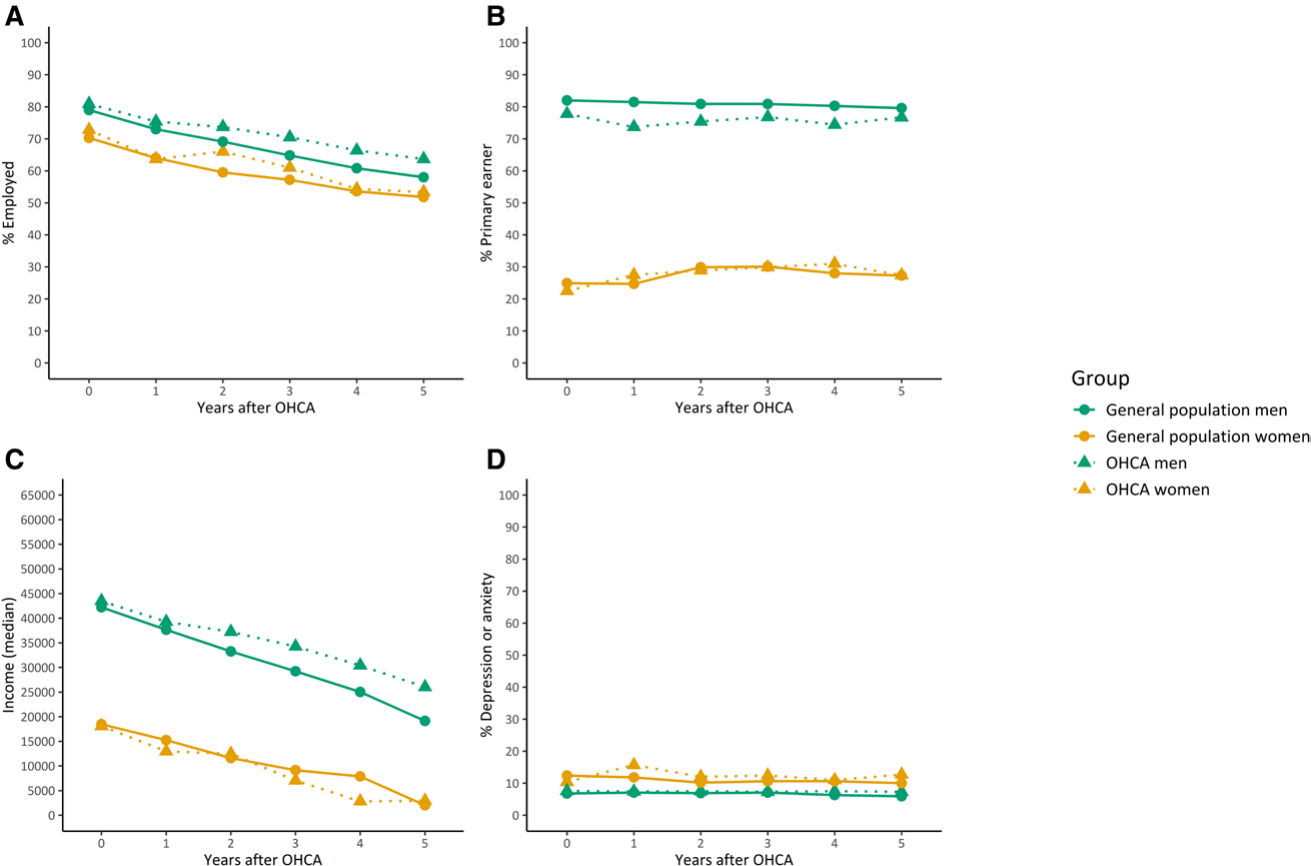
**Outcomes over time in OHCA patients and matched individuals, stratified by sex.** Panels display changes over time in: (**A**) employment rate; (**B**) primary earner rate; (**C**) median income; and (**D**) depression or anxiety medication. Employment and primary earner rates and median income were calculated in the study population aged ≤60 years. OHCA indicates out-of-hospital cardiac arrest.

In the general population, changes in outcomes appeared to be similar to the changes observed in the OHCA population (Figure). Statistically significant changes were observed in employment and income, but not in primary earner status (Table S3). The changes did not differ statistically significantly from those in OHCA patients (Table 3). However, in contrast to the small change observed in women after OHCA, proportions of anxiety/depression medication did not change in either sex in the general population. This difference between women from the OHCA and the general population was statistically significant (Table 3).

**Table 3.**
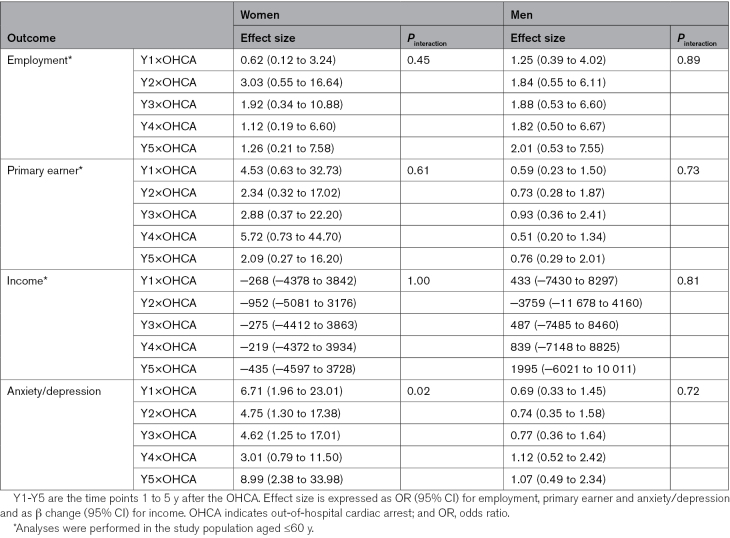
Interaction Between OHCA and Time, in Women and Men for Different Outcomes in a Combined Population of OHCA Patients and Matched Individuals Without OHCA

Sensitivity analysis with generalized estimating equations and the baseline of the outcomes defined based on values 2 years before OHCA yielded consistent findings to our main analyses (Tables S4 and S5).

## DISCUSSION

We investigated changes in socioeconomic and mental health outcomes in a Dutch community-based OHCA cohort over the first 5 years after OHCA. Employment and income decreased over this period in both women and men, while primary earner status (in those cohabiting) increased slightly in women but decreased slightly in men. The prevalence of anxiety/depression medication increased, but only in women. Only this change in prevalence of anxiety/depression medication differed from the patterns observed in the general population, suggesting that the increase may be specific to women who survive OHCA.

The strength of this study is the combination of an OHCA cohort with socioeconomic data from national registries. However, in the interpretation of these findings, several limitations should be considered. First, the outcome measures used in this study may have been suboptimal. The assessment of outcomes relied on the data available at the time of the research and was limited to yearly intervals, preventing a dynamic assessment of changes (eg, worsening or improving of symptoms within 1 year). For instance, the assessment of employment was dichotomous and based solely on tax reports of personal income generated from businesses and professions within the given fiscal year. The variable income used in our analysis was also based on tax reports. The intricacies of the Dutch system may result in an underestimation of the (temporary) effects of OHCA on income and employment, particularly in the first years after OHCA. The Netherlands offers a robust safety net to support people unable to work due to various reasons. If an individual experiences a work disability resulting from illness or injury, the employer is legally obliged to continue payment of at least 70% of the salary during the initial 2 years. Consequently, the manifestation of disability might not be immediately apparent in our selected outcome variables until 2 years after the occurrence of OHCA. However, the decline in income over all 5 years was consistent.

Furthermore, the classification of anxiety/depression was based on medication prescription records. Individuals who received other therapeutic interventions, for example, cognitive behavioral therapy, or individuals with untreated or undiagnosed anxiety/depression may have been missed. In addition, prescription rates are higher in women with diagnosed depression compared with men.^28^ Medication may reflect the willingness to take medications or the reporting of symptoms. Thus, we may have failed to capture the entirety of the emotional challenges experienced by survivors and overestimated differences between women and men, and caution in interpreting the observed differences is warranted.

Moreover, when OHCA survivors died within the follow-up period, they were excluded from subsequent analyses. As a consequence, the analysis predominantly consisted of relatively healthy survivors. This potentially leads to an underestimation of the impact of OHCA. On top of that, a different mortality rate in women and men would influence the comparison between sexes. Yet, our data did not indicate a sex difference in long-term mortality.

Finally, while we examined multiple outcomes, no correction for multiple testing was done.^25,29,30^ This approach was chosen because of our prioritization of investigating the 5-year changes in the OHCA population as our primary outcome. Applying a correction for multiple testing such as Bonferroni or Holm to the primary outcomes or the primary outcomes and the additional analyses would have affected the statistical significance of some of the associations. However, it does not change observed patterns and, thus, the general interpretation of our findings. Our findings should be confirmed in future studies.

Similar to previous studies, we observed that employment rates and income following OHCA were lower than before the OHCA^6,13^ and declined gradually over time. As opposed to our findings, income remained the same 1 year after OHCA compared with pre-OHCA in a Danish cohort.^6^ This discrepancy may be attributed to the composition of our analysis, which included people who do not work or return to work and thus have an income of zero, while the Danish study only included people who were employed before their OHCA and returned to work.

Despite the observed changes, we could not establish that the changes were likely related to the OHCA, as this pattern of decline in employment and income was also evident in the general population. This decrease in employment and income may relate in part to the early retirement of the people included in the study. According to Statistics Netherlands, a considerable amount of the Dutch population retires early. Notably, although the legal pension age was 65 years during the study period, between 6% and 8.8% of the 55+ population retired before that age.^31^ Multimorbidity plausibly plays a role, considering its rise after the age of 40,^32^ with potential associations between various chronic conditions and unemployment,^33–35^ and difficulty returning to work.^36^ However, we did not have data on comorbidities to explore this. Further research may unravel whether the reasons for this early retirement differed for those with OHCA versus the general population. Moreover, further work should consider broader aspects beyond changes in income and employment status alone. Re-entering the workforce or remaining employed does not necessarily indicate full recovery from OHCA and may mask aspects such as changes in working hours, changes in responsibilities, or job transitions. This may be different for women and men, as it may depend on the flexibility of the occupation that people work in or the availability of other resources, such as social support and household financial situations. Self-reported qualitative or quantitative data on barriers that people face and decisions they make regarding their socioeconomic position could provide valuable insight into OHCA survivors’ experiences in various domains and should be included in future work.^37^

Primary earner rates in our study were relatively stable; only a slight decrease in men and a small increase in women was observed, resulting in a modest sex difference in trends over time. This shift may relate to the fact that women participate in the workforce more often after their children have grown older.^38^ Moreover, men are generally older than women in relationships,^39^ increasing the likelihood of men retiring earlier than their partner, who is then more likely to become the primary earner. However, the observed patterns in women and men were similar to those in the general population. Such a general shift in primary earner status, even if modest, could have implications for societal roles and economic dynamics. The fulfillment of the role of primary earner is strongly gendered. Traditionally, men are the breadwinners of the family, and this is still the case in the majority of households.^40^

In our study, the change in anxiety/depression medication rates after OHCA differed between women and men. Particularly in women, an increase was observed. This is relevant given that depression is associated with mental quality of life^41^ and may be a risk factor for long-term mortality.^42^ In line with our study, another study reported higher levels of anxiety/depression after 4 months in women compared with men.^17,43^ Our study demonstrated that this change is present up to 5 years after OHCA. A previous study examined self-reported anxiety and depression and revealed a higher occurrence of these conditions than our study; one-third of patients reported these conditions over a period of 1 to 5 years.^44^ A possible explanation may be a difference in preexistent anxiety/depression within the study population, reflecting a higher prevalence within the study context. However, the change in rate in that study could not be determined, as a critical limitation was that pre-OHCA measurements for anxiety and depression were lacking. Besides, that study lacked a comparison with the general population, which hindered the ability to definitively attribute the observed prevalence of anxiety and depression solely to the OHCA event.

We show that the increase in anxiety/depression medication in women may be specific to OHCA survivors, as we did not observe a similar increase within the general population. Similar to our findings, another study found that anxiety and depression were high in both women and men in the first year after OHCA compared with people from the general population, but also compared with patients with myocardial infarction.^45^ The latter is important as it indicates that the difference is not likely caused by higher medication prescription rates related to intensified monitoring after an event (assuming that monitoring is similarly intensive postmyocardial infarction and post-OHCA).

Several potential explanations exist for the observed differences between women and men in changes in anxiety/depression after OHCA. The differences could stem from the divergent effects of OHCA on the brains of women and men. However, female sex hormones are considered to be neuroprotective,^46,47^ which seems to contradict our findings. Alternatively, the differences might be attributed to differences in sociocultural factors. Lower SES in women may explain the higher anxiety/depression rates.^23,48^ As a result of a low SES, income stress could potentially heighten the vulnerability to mental health issues and amplify the impact of OHCA. This may disproportionally impact women, as they typically have lower incomes.^23^ In our study, we observed that SES partially accounted for the sex difference in anxiety/depression changes. The increased rates of anxiety/depression in women who survived OHCA and the observed differences between women and men call for continued research efforts to increase the understanding of who is most affected and why. However, as stated in our limitations, it is possible that men less frequently seek help and that their willingness to take medication is lower. If so, changes in mental health may also be present in men who survived OHCA, but they may not be captured with a medication prescription.

We recommend that future studies into the long-term effects of OHCA include more extensive measures of anxiety/depression. Moreover, we recommend that such work be performed within different contexts and health care settings. Our findings may be dependent on the high quality of care in the Netherlands.^49^ Knowledge from the present study and these additional studies may ultimately stimulate the development of comprehensive and individualized interventions that enhance the mental health, and ultimately the well-being and quality of life, of all OHCA survivors.

Overall, the present study offers valuable insights into the socioeconomic and mental health changes over 5 years following OHCA. When compared with the general population, the observed decline in employment and income and a sex difference in trend in primary earner status did not seem drastic. However, future research should investigate the quality of the return to work and the causes underlying the decision not to return. Understanding whether unemployment or not returning to work is a personal choice or a consequence of an inability to work will guide health care professionals in providing appropriate support to these individuals. The observed increase in anxiety/depression among women who survived OHCA was stronger compared with the general population. This emphasizes the importance of addressing the mental health of OHCA survivors, specifically women, multiple years after the event.

## ARTICLE INFORMATION

### Acknowledgments

The authors thank all those contributing to the Amsterdam Resuscitation Studies study: participating emergency medical services dispatch centers (Amsterdam, Haarlem, and Alkmaar), regional ambulance services (Ambulance Amsterdam, GGD Kennemerland, Witte Kruis, Veiligheidsregio Noord-Holland Noord Ambulancezorg), fire brigades, police departments, and Schiphol airport, for their cooperation and support. We greatly appreciate the contributions of Vera van Eeden, Remy Stieglis, Mette Ekkel, Emma Linssen, and Rianne Kalk of the Academic Medical Centre (Amsterdam, The Netherlands) to the data collection, data entry, and patient follow-up.

### Sources of Funding

This study has received funding from the European Union’s Horizon 2020 research and innovation program under acronym European Sudden Cardiac Arrest network: towards Prevention, Education, New Effective Treatment, registered under grant agreement No. 733381, and the European Cooperation in Science and Technology (COST) Action Sudden cardiac arrest prediction and resuscitation network: Improving the quality of care (grant agreement No. CA19137) supported by COST, and the Netherlands CardioVascular Research Initiative, Dutch Heart Foundation, Dutch Federation of University Medical Centres, Netherlands Organization for Health Research and Development, and Royal Netherlands Academy of Sciences—CVON2017-15 RESCUED.

### Disclosures

None.

### Supplemental Material

Figure S1

Tables S1–S5

## Supplementary Material


