# Supplementary material for: Long-Term Socioeconomic and Mental Health Changes After Out-of-Hospital Cardiac Arrest in Women and Men
Source: Circ Cardiovasc Qual Outcomes. 2024 Jul 8;17(9):e011072. doi: 10.1161/CIRCOUTCOMES.124.011072 (PMC11415049; doi:10.1161/CIRCOUTCOMES.124.011072)
Supplement: Supplementary file 1 [file hcq-17-e011072-s001.pdf]

SUPPLEMENTAL MATERIAL

Figure S1. Flowchart of the study population

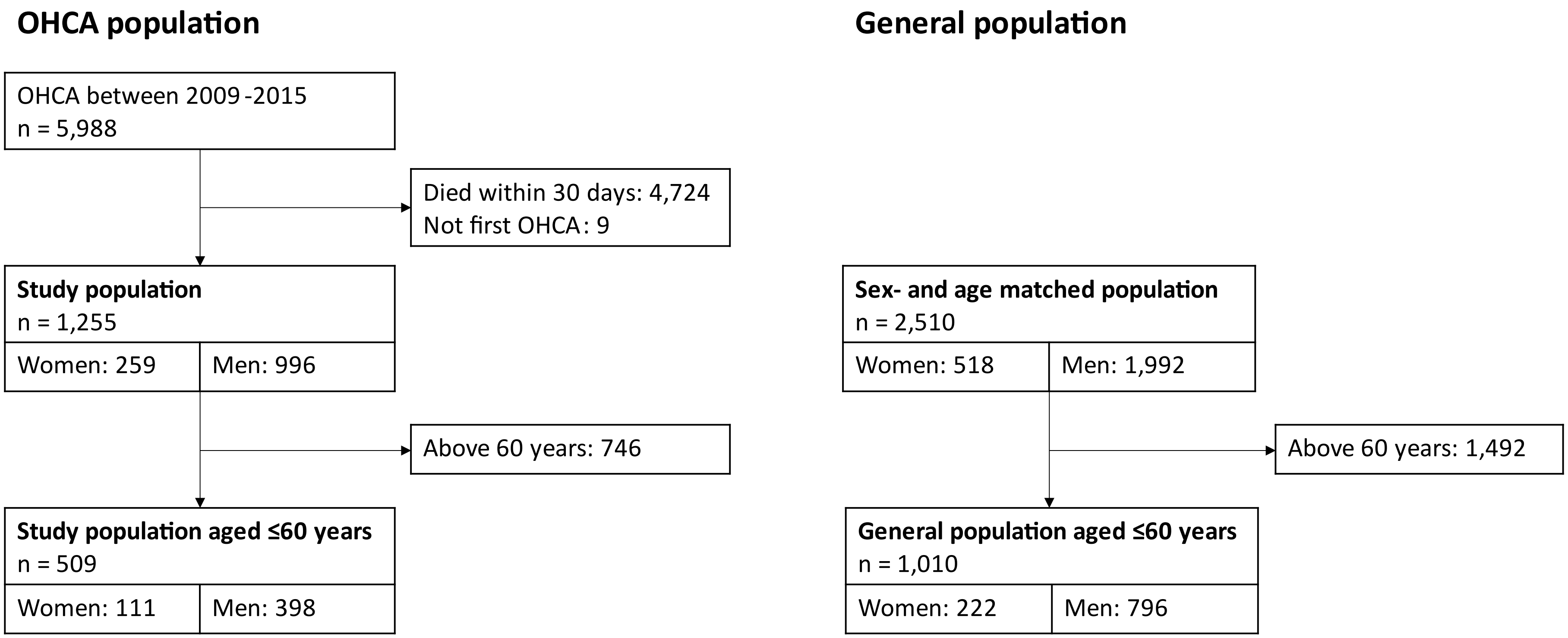

OHCA = out-of-hospital cardiac arrest

Table S1. Outcomes over time in the OHCA population overall and stratified by sex

	Baseline	Y1	Y2	Y3	Y4	Y5
**Women**						
	Baseline	Y1	Y2	Y3	Y4	Y5
Employment status*						
Employed	80 (72.8)	69 (63.7)	70 (66.0)	64 (61.0)	57 (54.3)	55 (53.4)
Unemployed	30 (27.2)	39 (36.3)	36 (34.0)	41 (39.0)	48 (45.7)	48 (46.6)
Missing	1	3	5	6	6	8
Income, median [IQR]*	18105 [0, 36467]	12962 [0, 34682]	12460 [0. 33811]	7068 [0, 33237]	2823 [0, 34428]	2933 [0, 35312]
Primary earner*						
Yes	20 (22.5)	25 (27.5)	26 (28.9)	26 (29.9)	27 (31.0)	23 (27.4)
No	69 (77.5)	66 (72.5)	6477.8 (71.1)	61 (70.1)	60 (69.0)	61 (72.6)
Missing	-	-	-	-	-	-
Anxiety/depression						
Yes	27 (10.4)	39 (15.7)	28 (12.0)	28 (12.4)	24 (11.0)	27 (12.7)
No	232 (89.6)	209 (84.3)	205 (88.0)	198 (87.6)	195 (89.0)	185 (87.3)
Missing	-	11	26	33	40	47
**Men**						
	Baseline	Y1	Y2	Y3	Y4	Y5
Employment status*						
Employed	321 (80.9)	295 (75.4)	280 (73.7)	261 (70.5)	245 (66.4)	232 (63.7)
Unemployed	76 (19.1)	96 (24.6)	100 (26.3)	109 (29.5)	124 (33.6)	132 (36.3)
Missing	1	7	18	28	29	34
Income, median [IQR]*	43486 [10140, 63830]	39266 [1, 61055]	37233 [0, 58966]	34252 [0, 58787]	30402 [0, 56339]	26023 [0, 58006]
Primary earner*						
Yes	249 (77.8)	233 (73.7)	233 (75.4)	228 (76.8)	216 (74.7)	214 (76.7)
No	71 (22.2)	83 (26.3)	76 (24.6)	69 (23.2)	73 (25.3)	65 (23.3)
Missing	-	-	-	-	-	-
Anxiety/depression						
Yes	76 (7.6)	70 (7.4)	68 (7.5)	64 (7.3)	63 (7.5)	60 (7.3)
No	920 (92.4)	876 (92.6)	837 (92.5)	808 (92.7)	782 (92.5)	763 (92.7)

Missing	-	50	91	124	151	173

Y1-Y5 are the timepoints 1 to 5 years after the OHCA. *Analyses were performed in the study population aged ≤60 years. IQR=interquartile range; OHCA=out-of-hospital cardiac arrest.

Table S2. Interaction between sex and time adjusted for household income

		Sex*time		Sex*time + household income		
**Outcome**	**Time**	**β**	**OR (95% CI)**	**β**		**% β change**
Primary earner*	Y1	-1.95	0.14 (0.02-0.84)	-1.96	0.14 (0.02-0.84)	0.6
	Y2	-2.52	0.08 (0.01-0.49)	-2.49	0.08 (0.01-0.51)	-1.1
	Y3	-2.73	0.06 (0.01-0.42)	-2.72	0.07 (0.01-0.43)	-0.7
	Y4	-3.36	0.03 (0.01-0.22)	-3.37	0.03 (0.01-0.22)	0.5
	Y5	-2.51	0.08 (0.01-0.52)	-2.51	0.08 (0.01-0.53)	-0.2
Anxiety/depression	Y1	-1.82	0.16 (0.05-0.53)	-1.57	0.21 (0.06-0.69)	-13.3
	Y2	-0.93	0.39 (0.11-1.36)	-0.82	0.44 (0.13-1.53)	-11.8
	Y3	-1.00	0.37 (0.11-1.29)	-0.89	0.41 (0.12-1.45)	-10.7
	Y4	-0.49	0.61 (0.17-2.23)	-0.39	0.68 (0.18-2.5)	-21.3
	Y5	-1.54	0.21 (0.06-0.77)	-1.47	0.23 (0.06-0.84)	-4.8

Y1-Y5 are the timepoints 1 to 5 years after the OHCA. *Analyses were performed in the study population aged ≤60 years. OR = odds ratio

Table S3. Changes in outcomes over time in the general population compared to baseline by the GLMM, stratified by sex, and interaction between sex and time

**Outcome**		**Women**		**Men**		
		Effect size	p-value	Effect size	p-value	$p_{interaction}$
Employment*	Y1	0.19 (0.06-0.59)	<0.001	0.10 (0.05-0.21)	<0.001	0.51
	Y2	0.05 (0.01-0.18)		0.03 (0.02-0.07)		
	Y3	0.02 (0.01-0.10)		0.01 (0.005-0.02)		
	Y4	0.01 (0.003-0.06)		0.003 (0.001-0.01)		
	Y5	0.01 (0.002-0.04)		0.002 (0.001-0.004)		
Primary earner*	Y1	0.77 (0.26-2.30)	0.05	0.82 (0.46-1.43)	0.20	0.01
	Y2	2.68 (0.93-7.73)		0.67 (0.38-1.17)		
	Y3	3.34 (1.16-9.62)		0.62 (0.35-1.09)		
	Y4	1.82 (0.61-5.39)		0.66 (0.37-1.16)		
	Y5	2.21 (0.73-6.67)		0.48 (0.27-0.86)		
Income*	Y1	-1892 (-4312, 528)	<0.001	-3111 (-8516, 2294)	<0.001	0.69
	Y2	-3097 (-5517, -678)		-1386 (-6806, 4034)		
	Y3	-4634 (-7054, -2215)		-7368 (-12809, -1928)		
	Y4	-5155 (-7589, -2720)		-9981 (-15440, -4521)		
	Y5	-5510 (-7944, -3075)		-11207 (-16682, -5731)		
Anxiety/depression	Y1	0.88 (0.44-1.76)	0.69	1.40 (0.91-2.15)	0.28	0.34
	Y2	0.56 (0.27-1.15)		1.41 (0.91-2.18)		
	Y3	0.75 (0.37-1.54)		1.67 (1.08-2.58)		
	Y4	0.77 (0.38-1.57)		1.26 (0.81-1.97)		
	Y5	0.66 (0.32-1.37)		1.20 (0.76-1.88)		

Y1-Y5 are the timepoints 1 to 5 years after the OHCA. Effect size is expressed as OR (95%CI) for employment, primary earner and anxiety/depression and as β change (95% CI) for income. *Analyses were performed in the study population aged ≤60 years. CI = confidence interval; GLMM = generalized linear mixed model; OR = odds ratio.

Table S4. Result of the GEE for changes in outcome over time, stratified by sex

Outcome		Women		Men		
		Effect size	p-value	Effect size	p-value	$p_{interaction}$
Employment*	Y1	0.64 (0.48-0.86)	<0.001	0.71 (0.60-0.84)	<0.001	0.64
	Y2	0.69 (0.50-0.97)		0.61 (0.51-0.74)		
	Y3	0.54 (0.37-0.78)		0.50 (0.40-0.62)		
	Y4	0.41 (0.27-0.62)		0.40 (0.32-0.50)		
	Y5	0.39 (0.26-0.58)		0.36 (0.28-0.45)		
Primary earner*	Y1	1.23 (0.86-1.77)	0.13	0.80 (0.65-0.98)	0.19	0.08
	Y2	1.37 (1.01-1.86)		0.83 (0.66-1.04)		
	Y3	1.47 (1.06-2.04)		0.87 (0.70-1.10)		
	Y4	1.49 (1.00-2.24)		0.79 (0.62-1.01)		
	Y5	1.30 (0.86-1.97)		0.90 (0.70-1.16)		
Income*	Y1	-2166 (-4179, -153)	0.07	-2592 (-4956, -228)	<0.001	0.47
	Y2	-4050 (-7025, -1074)		-5287 (-8228, -2345)		
	Y3	-4902 (-8549, -1255)		-7248 (-10562, -3934)		
	Y4	-5373 (-9078, -1669)		-9502 (-12855, -6150)		
	Y5	-5932 (-10802, -1063)		-9584 (-13271, -5896)		
Anxiety/depression	Y1	1.68 (1.14-2.46)	0.07	0.98 (0.77-1.26)	0.96	0.13
	Y2	1.31 (0.89-1.92)		1.01 (0.80-1.27)		
	Y3	1.41 (0.97-2.05)		1.06 (0.82-1.37)		
	Y4	1.25 (0.87-1.80)		1.09 (0.84-1.41)		
	Y5	1.62 (1.08-2.43)		1.06 (0.82-1.36)		

Y1-Y5 are the timepoints 1 to 5 years after the OHCA. Effect size is expressed as OR (95%CI) for employment, primary earner and anxiety/depression and as β change (95% CI) for income. *Analyses were performed in the study population aged ≤60 years. CI = confidence interval; GEE = generalized estimating equations; OR = odds ratio.

Table S5. Result of the GLMM for changes in outcome over time with 2 years before OHCA as baseline, stratified by sex

**Outcome**		**Women**		**Men**		
		Effect size	p-value	Effect size	p-value	$p_{interaction}$
Employment*	Y1	0.06 (0.01-0.25)	<0.001	0.08 (0.03-0.23)	<0.001	0.43
	Y2	0.08 (0.02-0.33)		0.04 (0.01-0.11)		
	Y3	0.03 (0.01-0.14)		0.01 (0.004-0.04)		
	Y4	0.01 (0.007-0.06)		0.003 (0.001-0.01)		
	Y5	0.01 (0.002-0.05)		0.002 (0.001-0.01)		
Primary earner*	Y1	1.85 (0.42-8.16)	0.34	0.37 (0.18-0.79)	0.009	N/A
	Y2	3.11 (0.7-13.88)		0.39 (0.18-0.83)		
	Y3	4.40 (0.94-20.58)		0.44 (0.20-0.95)		
	Y4	4.67 (1.01-21.67)		0.26 (0.12-0.57)		
	Y5	2.38 (0.51-11.15)		0.29 (0.13-0.64)		
Income*	Y1	-2084 (-5279, 1111)	0.003	-3803 (-6214, -1392)	<0.001	0.24
	Y2	-3968 (-7185, -751)		-6530 (-8966, -4094)		
	Y3	-4818 (-8045, -1592)		-8513 (-10970, -6055)		
	Y4	-5286 (-8521, -2052)		-10769 (-13229, -8310)		
	Y5	-5844 (-9089, -2598)		-10866 (-13335, -8396)		
Anxiety/depression	Y1	1.77 (0.75-4.15)	0.43	1.18 (0.64-2.18)	0.74	0.48
	Y2	0.94 (0.38-2.34)		1.20 (0.65-2.24)		
	Y3	1.15 (0.46-2.89)		1.45 (0.78-2.72)		
	Y4	0.81 (0.31-2.12)		1.60 (0.85-3.01)		
	Y5	1.75 (0.69-4.45)		1.43 (0.75-2.72)		

Y1-Y5 are the timepoints 1 to 5 years after the OHCA. Effect size is expressed as OR (95%CI) for employment, primary earner and anxiety/depression and as β change (95% CI) for income. *Analyses were performed in the study population aged ≤60 years. CI = confidence interval; GLMM = generalized linear mixed model; OHCA = out-of-hospital cardiac arrest; OR = odds ratio.
